# Supplementary material for: A comparative in-silico analysis of autophagy proteins in ciliates
Source: PeerJ. 2017 Jan 17;5:e2878. doi: 10.7717/peerj.2878 (PMC5244887; doi:10.7717/peerj.2878)
Supplement: Supplemental Information 1 [file peerj-05-2878-s001.docx]

***Supplemental Information***

**A Comparative in-silico analysis of autophagy proteins in ciliates**

Erhan Aslan^1,2^, Nurçin Küçükoğlu^1,2^ and Muhittin Arslanyolu^2^

^1^Graduate School of Science, Department of Molecular Biology, Anadolu University, 26470, Eskişehir, Turkey

^2^Laboratory of Molecular Biotechnology and Enzymology, Faculty of Science, Department of Biology, Anadolu University, 26470, Eskişehir, Turkey.

**Table S1**. **List of accession numbers of autophagy proteins in ciliates**.

| Proteins | *Tetrahymena*  *thermophila* | *Paramecium tetraurelia* | *Oxytricha trifallax* | *Ichthyophthirius multifiliis* | *Stylonychia lemnae* |
| --- | --- | --- | --- | --- | --- |
| Atg1  Atg2  Atg3  Atg4  Atg5  Atg6  Atg7  Atg8  Atg9  Atg10  Atg11  Atg12  Atg13  Atg14  Atg15  Atg16  Atg16L  Atg18  Vps15  Vps34  Vmp-1  UVRAG | nd  nd  TTHERM_01050620  TTHERM_00012980  TTHERM_00622890  TTHERM_00011010  TTHERM_00526270  TTHERM_00622880  TTHERM_00011020  TTHERM_00494030  TTHERM_000455639  TTHERM_00138370  TTHERM_00522490  TTHERM_00037460  TTHERM_000780499  nd  TTHERM_01016200  nd  nd  nd  nd  TTHERM_00691620  nd  nd  TTHERM_00614790  TTHERM_00577340  TTHERM_00543659  TTHERM_00649380  TTHERM_00628720  nd | nd  nd  GSPATT00018613001  GSPATT00000653001  GSPATT00032381001  GSPATT00027262001  GSPATT00009966001  GSPATT00016554001  GSPATT00003976001  GSPATT00007114001  GSPATT00029795001  GSPATT00039312001  GSPATT00000189001  GSPATT00008726001  GSPATT00037390001  GSPATT00008226001  GSPATT00012815001  GSPATT00013195001  GSPATT00019394001  GSPATT00019297001  GSPATT00015805001  GSPATT00027839001  GSPATT00002599001  GSPATT00039589001  GSPATT00001448001  GSPATT00022734001  GSPATT00015140001  GSPATT00002727001  GSPATT00001312001  GSPATT00037476001  GSPATT00029699001  GSPATT00004799001  GSPATT00007583001  GSPATT00033828001  GSPATT00011139001  GSPATT00031859001  GSPATT00028409001  GSPATT00020835001  GSPATT00037728001  GSPATT00004842001  GSPATT00016042001  GSPATT00009773001  nd  nd  nd  nd  nd  nd  GSPATT00021619001  GSPATT00027425001  nd  nd  GSPATT00022741001  GSPATT00029706001  GSPATT00038932001  GSPATT00026656001  GSPATT00022039001  GSPATT00034458001  GSPATT00031445001  nd | nd  nd  OXYTRI_06717  OXYTRI_00763  OXYTRI_06906  OXYTRI_20407  OXYTRI_17161  OXYTRI_01553  OXYTRI_23932  OXYTRI_18949  OXYTRI_17764  OXYTRI_06903  OXYTRI_24789  OXYTRI_03397  OXYTRI_04222  OXYTRI_02617  OXYTRI_06951  nd  nd  nd  OXYTRI_18637  nd  nd  nd  nd  OXYTRI_20923  OXYTRI_00719  OXYTRI_14811  OXYTRI_14403  OXYTRI_14036  OXYTRI_18883  OXYTRI_23250  OXYTRI_00310  OXYTRI_13489 | nd  nd  IMG5_148300  IMG5_143150  IMG5_021070  IMG5_195560  IMG5_027770  nd  IMG5_184550  nd  nd  nd  nd  nd  nd  nd  nd  nd  nd  nd  IMG5_194800  IMG5_142140  nd  IMG5_193880  IMG5_198170  IMG5_083720  nd | nd  nd  STYLEM_5006  STYLEM_548  STYLEM_8437  STYLEM_6052  STYLEM_7592  STYLEM_8080  STYLEM_127  STYLEM_8328  STYLEM_1555  STYLEM_17224  STYLEM_3514  STYLEM_13036  nd  nd  nd  nd  nd  nd  nd  nd  nd  STYLEM_5216  STYLEM_15702  STYLEM_6597  STYLEM_15893  STYLEM_561  STYLEM_20251  STYLEM_9113  nd |
|  |  |  |  |  |  |

nd: not determined
