# Supplementary figures and images for: A comparative in-silico analysis of autophagy proteins in ciliates
Source: PeerJ. 2017 Jan 17;5:e2878. doi: 10.7717/peerj.2878 (PMC5244887; doi:10.7717/peerj.2878)

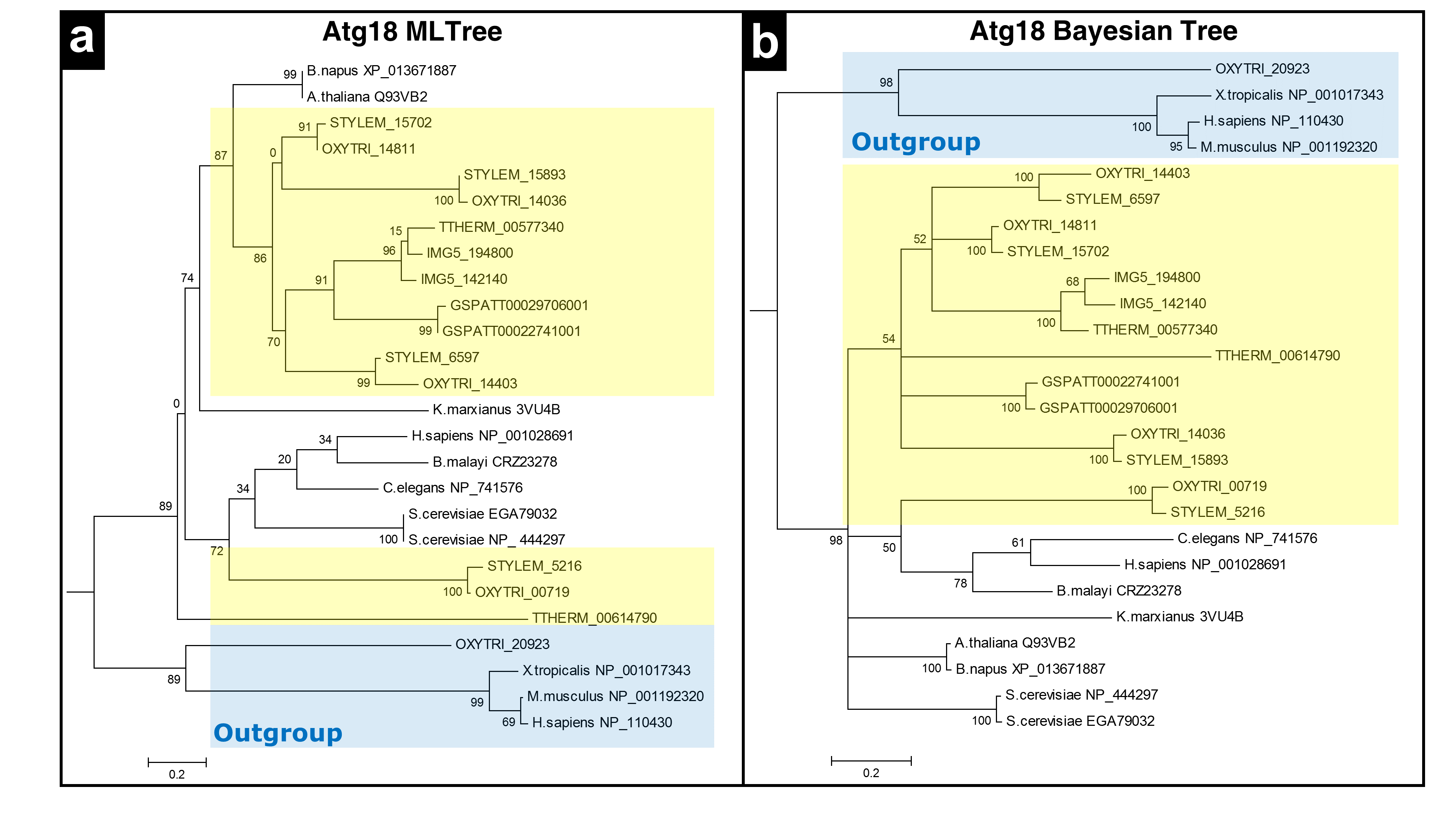

Supplement: Figure S1 — (A) Maximum likelihood (ML) tree. (B) Bayesian tree. Trees were computed based on multiple alignment of WD40 domains of Atg18 and Atg16L proteins. Since Atg16L proteins contain WD40 repeats like Atg18 proteins, trees were rooted with WD40 domain of a set of Atg16L proteins including one from Oxytricha (OXYTRI_209239) (blue box). Ciliate members were highlighted with light orange color. Both trees show the same placement of critical nodes. [file peerj-05-2878-s002.png]

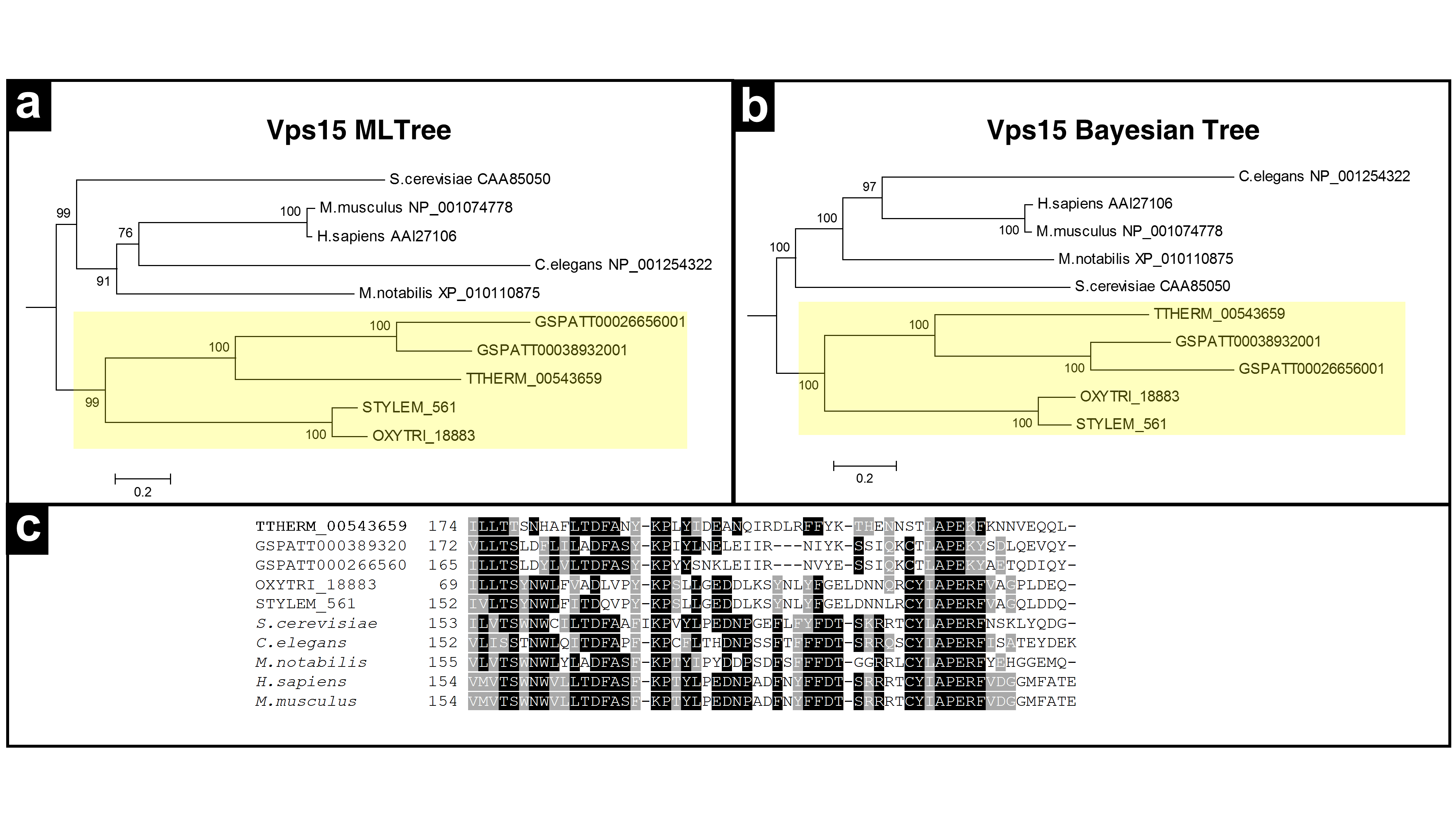

Supplement: Figure S2 — (A) Maximum likelihood (ML) tree. (B) Bayesian tree. Ciliate members were highlighted. Both trees show the same placement of critical nodes. (C) Partial multiple alignment of Vps15 proteins from ciliates and various model organisms. [file peerj-05-2878-s003.png]

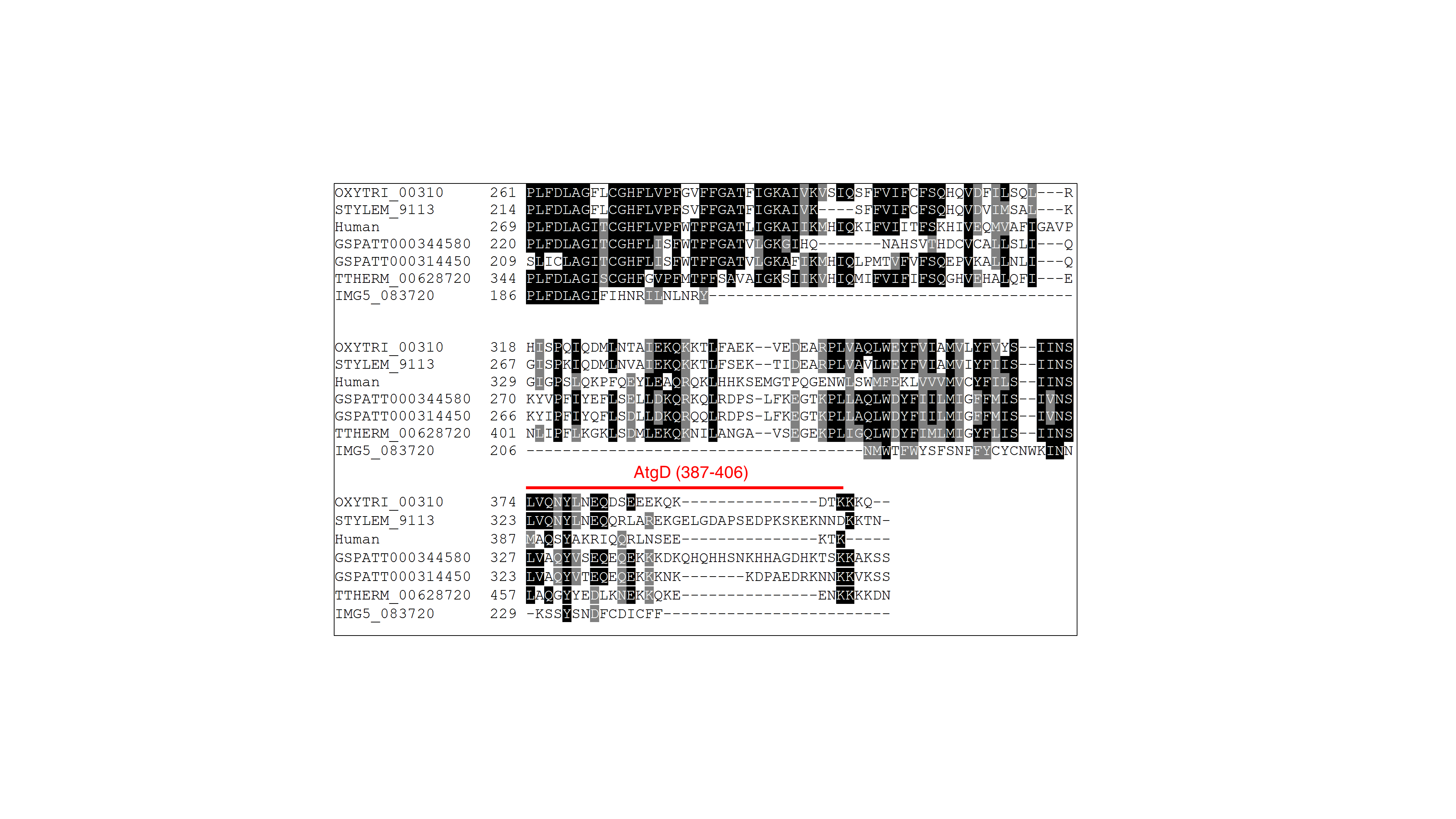

Supplement: Figure S3 — It has been shown that human Vmp1 protein (CAG38552.1) interacts with Beclin-1 through its C-terminal located autophagy (AtgD) domain to regulate autophagy induction (Molejon & Ropolo, 2013). AtgD domain of Human Vmp1 (387-406) is marked with red line. [file peerj-05-2878-s004.png]

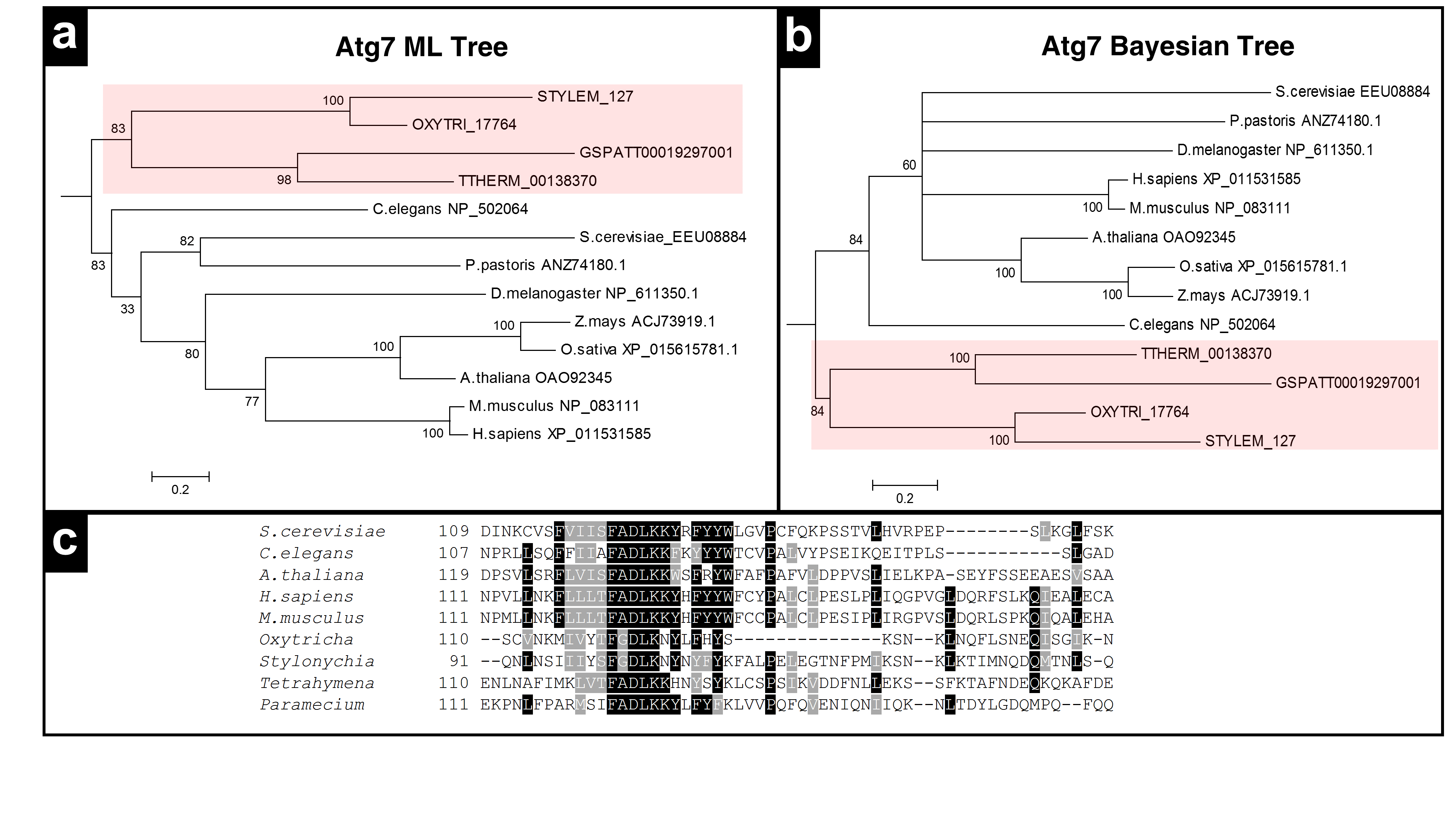

Supplement: Figure S4 — (A) Maximum likelihood (ML) tree. (B) Neighbor joining (NJ) tree. Trees were computed based on multiple alignment of N-terminal Atg7 domains. Both trees show the same placement of critical nodes. Ciliate members were highlighted with pink color (C) Partial multiple alignment of Atg7 proteins from ciliates and various model organisms. [file peerj-05-2878-s005.png]

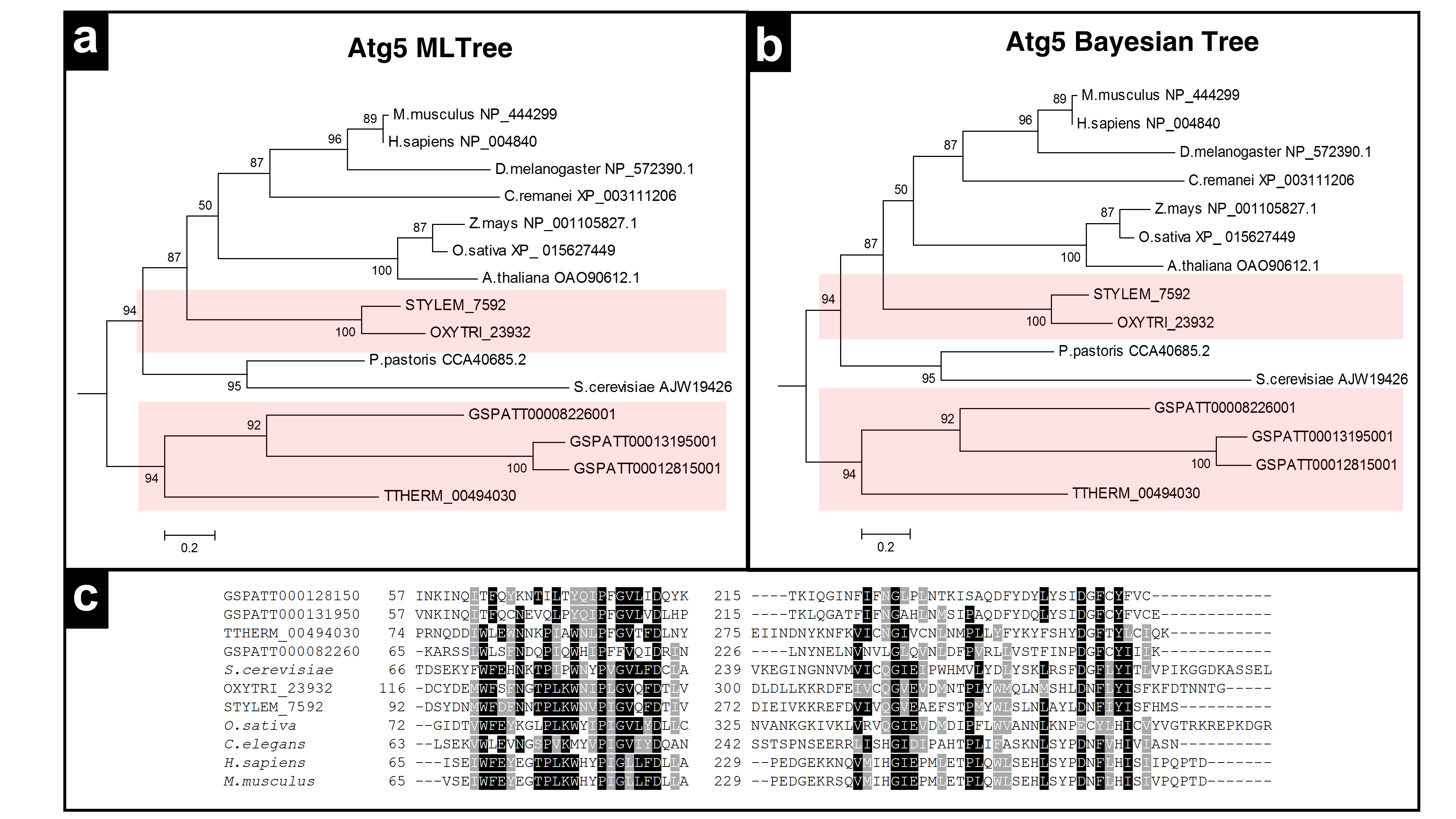

Supplement: Figure S5 — (A) Maximum likelihood (ML) tree. (B) Bayesian tree. Trees were computed based on multiple alignment of Atg5 domains. Ciliate members were highlighted with pink color. Both trees show the same placement of critical nodes. (C) Partial multiple alignment of Atg5 proteins from ciliates and various model organisms. [file peerj-05-2878-s006.png]

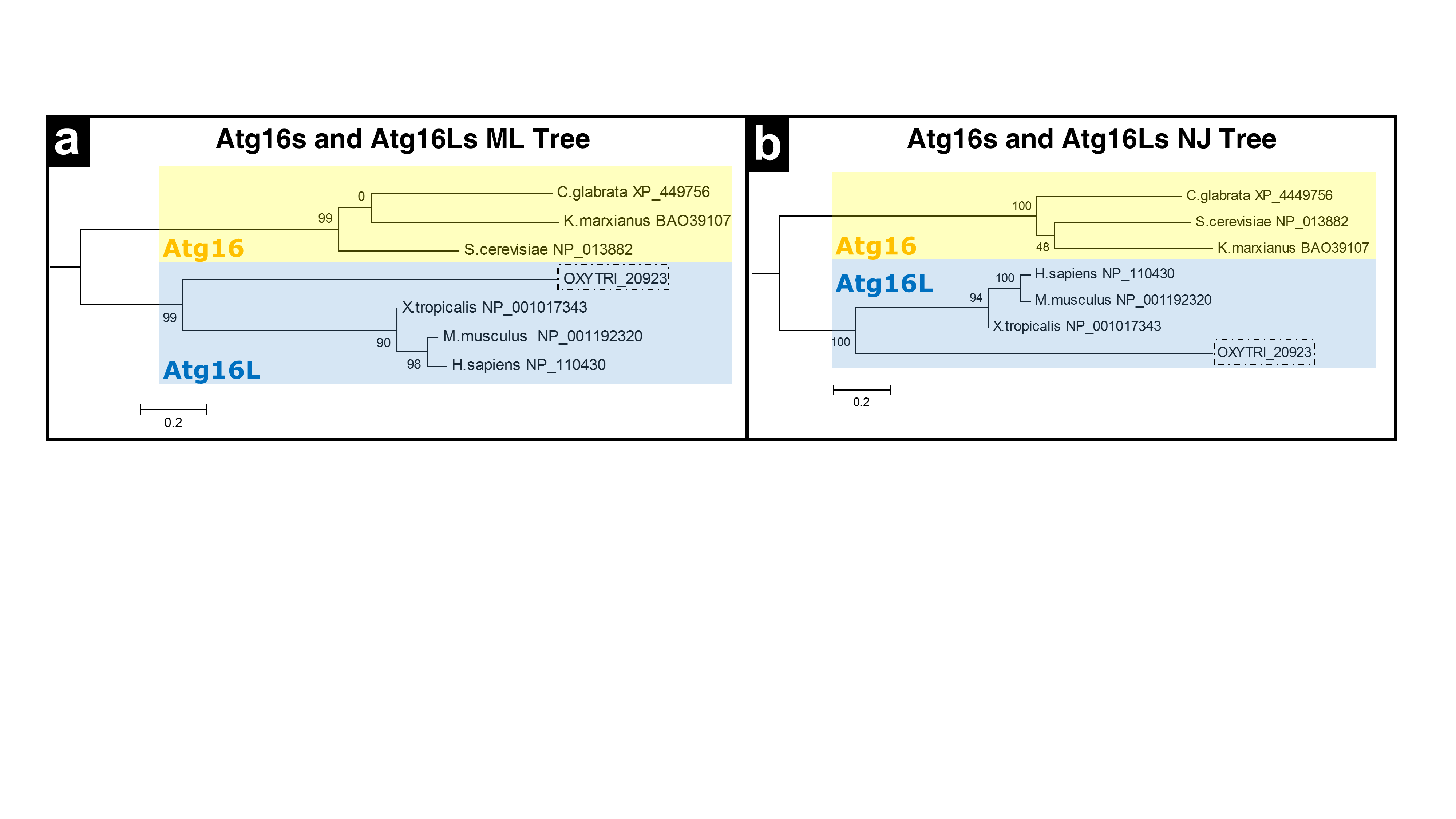

Supplement: Figure S6 — Phylogenetic analyses of Atg16s and Atg16Ls. (A) Maximum likelihood (ML) tree. (B) Neighbor joining tree. Trees were computed based on multiple alignment of Atg16 domains of yeast Atg16s and mammalian and reptile Atg16s. Both trees show that OXYTRI_20923 from Oxytricha (dotted box) is an Atg16L ortholog not Atg16. [file peerj-05-2878-s007.png]

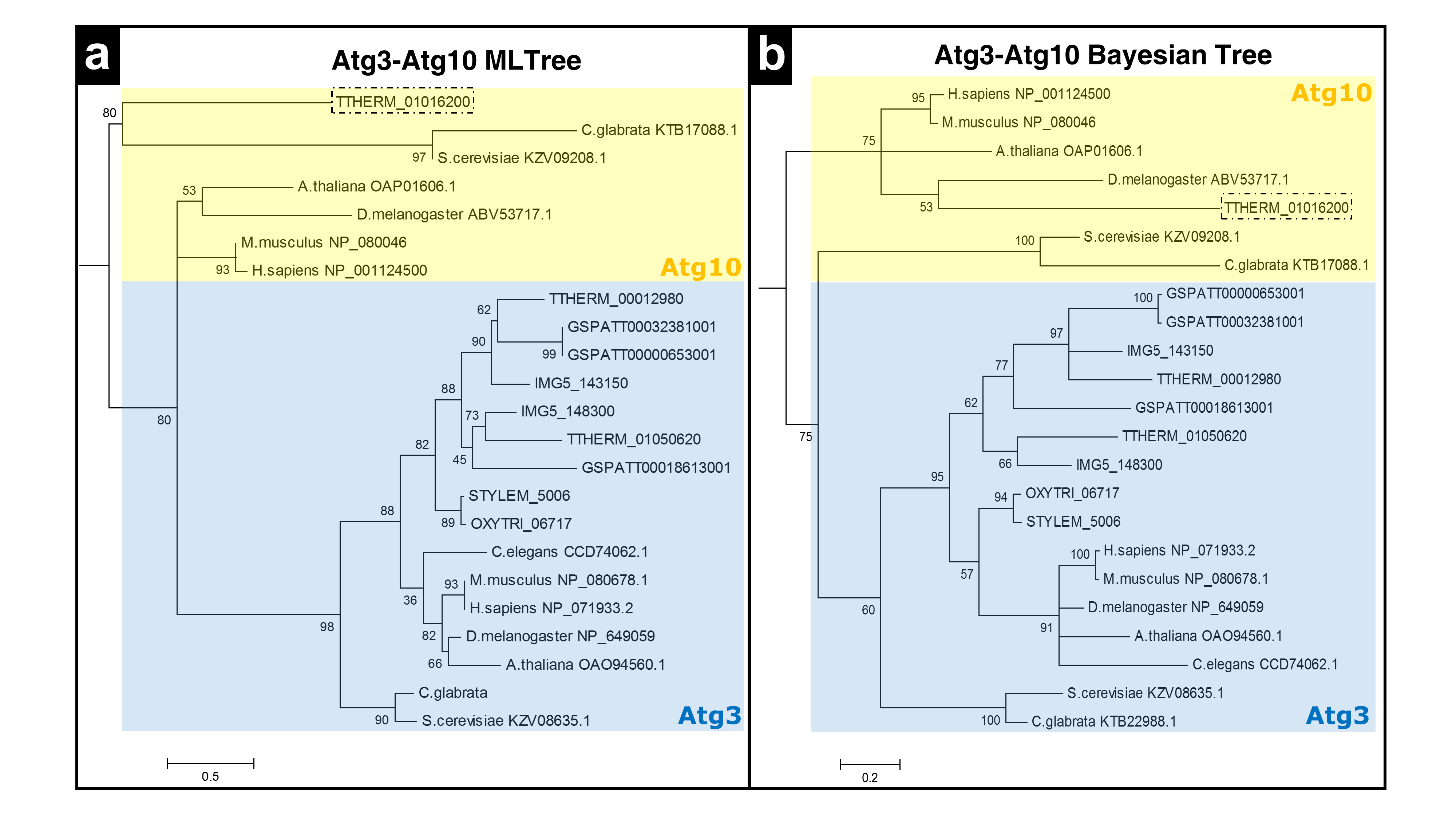

Supplement: Figure S7 — Phylogenetic analyses of Atg3s and Atg10s. (A) Maximum likelihood (ML) tree. (B) Bayesian tree. Trees were computed based on multiple alignment of Autophagy_act_C domains (PF03987) of Atg3s and Atg10s. Both trees show that TTHERM_00012980 from Tetrahymena (dotted box) is the sole Atg10 ortholog in ciliates. [file peerj-05-2878-s008.png]
